# Compositional MRI of the anterior cruciate ligament of professional alpine ski racers: preliminary report on seasonal changes and load sensitivity

**DOI:** 10.1186/s41747-020-00191-0

**Published:** 2020-11-23

**Authors:** Robert Csapo, Vladimir Juras, Bernhard Heinzle, Siegfried Trattnig, Christian Fink

**Affiliations:** 1 Research Unit for Orthopaedic Sports Medicine and Injury Prevention, ISAG, University for Health Sciences, Medical Informatics and Technology, Hall, A-6060 Austria; 2 Highfield MR Center, Department of Biomedical Imaging and Image-Guided Therapy, Medical University of Vienna, Lazarettgasse 14, A-1090 Vienna, Austria; 3 Kursana Private Hospital, Wörgl, A-6030 Austria; 4 CD Laboratory for Molecular Clinical MR Imaging, Vienna, Austria; 5 Gelenkpunkt Sports and Joint Surgery, Innsbruck, A-6020 Austria

**Keywords:** Athletes, Anterior cruciate ligament, Magnetic resonance imaging, Skiing, Seasons

## Abstract

The purpose of this study was to investigate potential changes in the anterior cruciate ligament (ACL) structure of alpine ski racers over the course of an entire season using quantitative magnetic resonance imaging (T2* mapping). The dominant legs of three alpine ski racers were examined on a 3-T MR scanner four times at 3-month intervals. Multi-echo sequences for T2* maps, which were coregistered with high-resolution morphological sequences for reproducible definition of ACL regions of interest, were acquired. Means and standard deviations of T2* values from the central and femoral portion of the ACL were extracted and presented in a descriptive manner. T2* values were subject to seasonal changes, which were most pronounced in the ligament central region. Substantial increases (+ 41%) occurred between the measurements taken in January and April. A partial recovery of T2* (-19%) was observed in the July follow-up. The increased T2* times may reflect decreased stress tolerance and increased susceptibility for fatigue tears at the end of the competitive season. Further research in larger samples is required. The likeliness of ACL tears may depend on the precedent history of mechanical loading and vary in professional athletes over the course of the competitive season.

## Key points

- Quantitative magnetic resonance imaging can detect seasonal changes in cruciate ligaments of ski racers.
- Anterior cruciate ligament (ACL) T2* values peaked in April at the end of season.
- ACL T2* increase may reflect a decreased stress tolerance increasing the risk of fatigue tears.

## Background

Tears of the anterior cruciate ligament (ACL) represent one of the most frequent musculoskeletal injuries and typically affect young and athletic populations [1, 2]. In alpine ski racing, a discipline characterised by high speeds and large forces imposed on the lower limbs, ACL tears are particularly common, with epidemiological data from the International Ski Federation Injury Surveillance System indicating an incidence of 5.4–8.5 injuries per 100 skier-seasons [3, 4]. While the typical mechanisms of ACL injury in alpine ski racing, namely the dynamic snow plow, slip catch, and back-weighted landing [5], are well described, observations of ACL tears occurring consequent to apparently minor stresses (and often not even resulting in crashes) raise questions about possible intrinsic factors predisposing athletes to injury.

Yet another parameter potentially associated with ACL injury risk is the precedent history of ACL loading. *In vitro* studies using cadaveric limbs have shown that repeated exposure to submaximal knee loading may alter the ACL stress tolerance, leading to sudden tear [6, 7]. Anecdotal evidence derived from the database of our sports clinic further suggests that the number of ACL reconstructions performed in professional alpine ski racers accumulates in the later stages of the competitive season. While this observation may be caused by a number of factors unrelated to the ACL mechanical properties, such as changes in environmental and snow conditions and neuromuscular or cognitive fatigue, alterations in the ligament’s microstructural and biochemical properties promoting fatigue tears cannot be ruled out.

One approach to obtaining imaging-based biomarkers indicative of the ACL structure and biochemistry is through magnetic resonance imaging (MRI)-based T2* mapping. As is well-known, T2* values reflect the decay of transverse magnetisation caused by spin-spin relaxation and magnetic field inhomogeneities:

$$ \frac{1}{T{2}^{\ast }}=\frac{1}{T2}+\gamma \Delta B $$

where T2 is the “true” transverse relaxation time, γ is the gyromagnetic ratio, and ΔB reflects the magnetic field inhomogeneities across a voxel [8]. T2* values reflect inherent tissue properties that are related to tissue composition (*e.g*., collagen fibre and water content) and organisation. In collagenous tissues, shorter T2* relaxation times have been found to be positively correlated to a more highly organised (*i.e.*, healthier) structure of menisci [9] and ligaments [10], whereas in cartilage T2* values are reportedly lower in degraded as compared to healthy tissue [11]. Thus, T2* maps may provide region-specific information about the integrity of different collagenous tissues, including the ACL [12].

The purpose of the T2* measurements in addition to conventional morphological MRI reported in this manuscript was to investigate potential changes in the ACL structure of alpine ski racers over the course of an entire season.

## Methods

Four male professional alpine skiers engaged at the European and World Cup level volunteered to participate in the study. One athlete suffered an ACL tear after the first measurement, so the study could be completed in three subjects, aged 23.3, 21.3, and 21.4 years, weighing 86, 80, and 76 kg, with a height of 182, 180, and 180 cm, respectively. All subjects participated exclusively in technical events (slalom and giant slalom). The participants were scheduled for four visits evenly distributed over the 2018–2019 season (appointments in October 2018, January 2019, April 2019, and July 2019). The study was approved by the ethics committee of the Medical University of Innsbruck (vote AN2016-0172), and all participants gave written informed consent.

### Imaging protocol

**Fig. 1:**
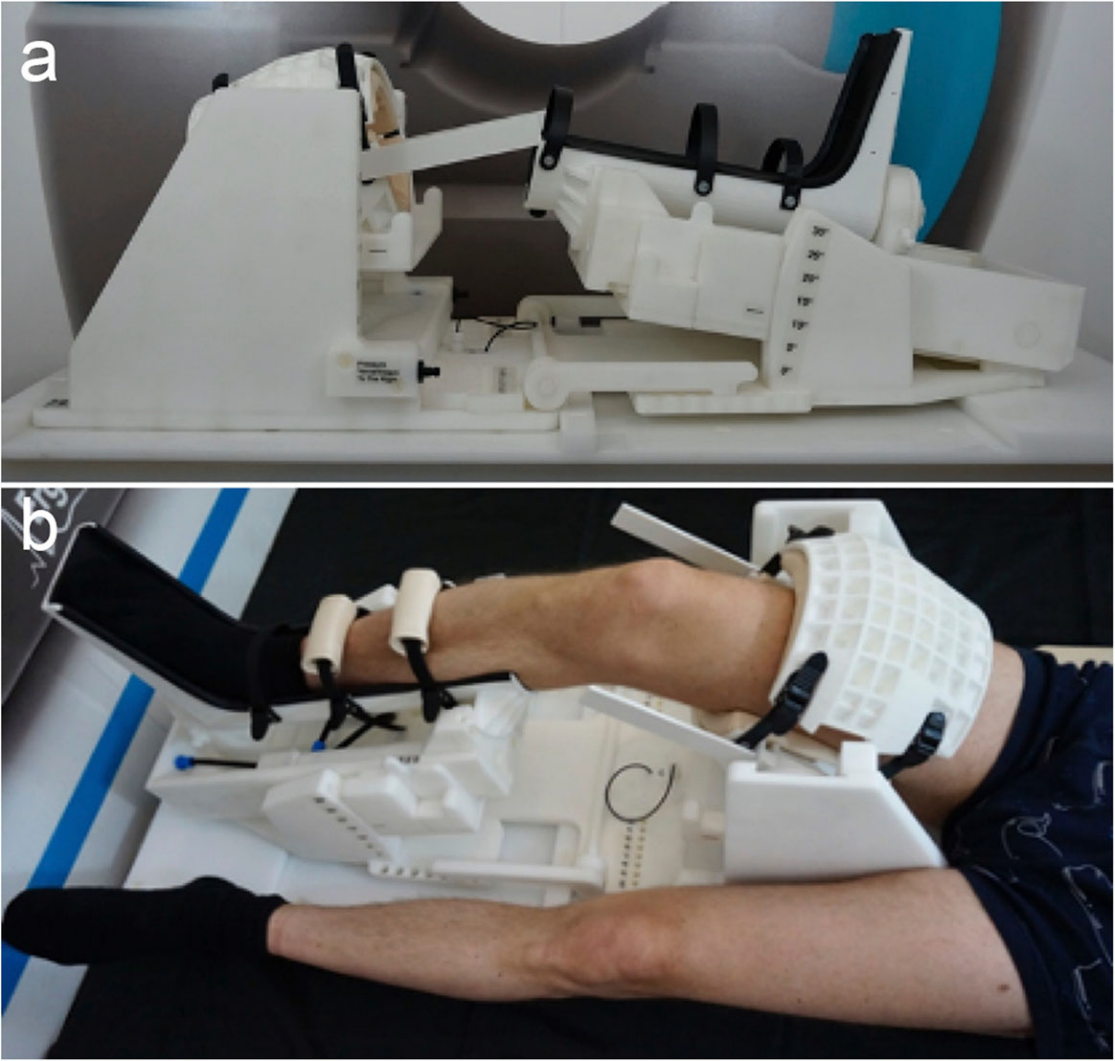
**a** MRI-safe, pneumatically driven knee fixation device. The “boot” of the device may be rotated upwards, to exert pressure against the posterior aspect of the calf, with the thigh being firmly anchored. This creates a Lachman test-like scenario in which the anterior cruciate ligament is strained. **b** Subject positioning within the device

Images were acquired at a private hospital on a 3-T whole-body scanner (Skyra, Siemens Healthineers, Erlangen, Germany) using a 6-channel flex coil. Subjects were positioned supine, with the knee of the dominant leg, defined as the leg preferentially used to kick a ball, fixed in an MRI-safe pneumatic knee fixation device at 10° of articular flexion (Fig. 1).

The MRI protocol consisted of the following consecutive sequences:

1. A high-resolution, fat-saturated proton density (PD)-weighted, turbo spin echo (TSE) sequence along a parasagittal plane;
2. A high-resolution isotropic, fat-saturated, isotropic PD-weighted sampling perfection with application of optimised contrasts using different flip angle evolution (PD SPACE) sequence, acquired at rest along a parasagittal plane;
3. A multi-echo T2*-weighted gradient-echo (GRE) sequence to measure T2* relaxation values;
4. A high-resolution isotropic, fat-saturated, isotropic PD SPACE sequence, as described at point 2;
5. A multi-echo T2*-weighted GRE sequence, as described at point 3.

Details regarding technical parameters of these MRI sequence are reported in Table 1.

**Table 1:** The MR sequence parameters

	Proton density-weighted	Proton density-weighted	T2*-weighted
Type	Turbo spin-echo	SPACE	Gradient echo
Fat saturation	Yes	Yes	No
Time of repetition (ms)	2,230	700	575
Time of echo (ms)	33	30	3.71, 10.56, 17.99, 25.42, 32.85, 40.28
Echo train length	7	23	-
Bandwidth (Hz/pixel)	250	385	260
In-plane resolution (mm × mm)	0.42 × 0.42	0.66 × 0.66	0.56 × 0.56
Through-plane resolution (mm)	2	0.70	2

*SPACE* Sampling perfection with application of optimised contrasts using different flip angle evolutions

First, sequences 1, 2, and 3 were acquired at rest. Then, the pneumatic system within the knee fixation device was used to apply 2.5 bar of pressure, equivalent to a force of 150 N, against the posterior aspect of the calf in order to provoke a Lachman test-like scenario, pushing the tibia against the fixed femur in the anterior direction and, thus, straining the ACL. During ongoing pressure application, sequences 4 and 5 were completed.

### Image postprocessing and evaluation

Using the multi-echo images obtained with sequences 3 and 5, the T2* relaxation times were estimated by fitting a monoexponential decay function to the signal intensities (SI) of voxels that decrease with increasing time of echo (TE):

$$ SI(TE)={S}_0{e}^{-\frac{TE}{T_2^{\ast }}} $$

To analyse the resultant T2* values, regions of interest (ROIs) were placed over the central and femoral region of the ACL. The tibial third of the ACL was deliberately omitted as measurements obtained in this region were considered to be most prone to bias related to focally increased signal intensities, which were physiologically implausible and presumably due to field orientation (also known as magic angle) artefacts [13]. To warrant consistent in-plane ROI positioning across pressure conditions and measurement points, automated image coregistration was employed. For this purpose, the high-resolution three-dimensional TSE sequence was aligned to T2* maps by cubic interpolation, making use of the image orientation information provided in the DICOM header. Then, corresponding high-resolution slices were selected in order to match with T2* maps.

**Fig. 2:**
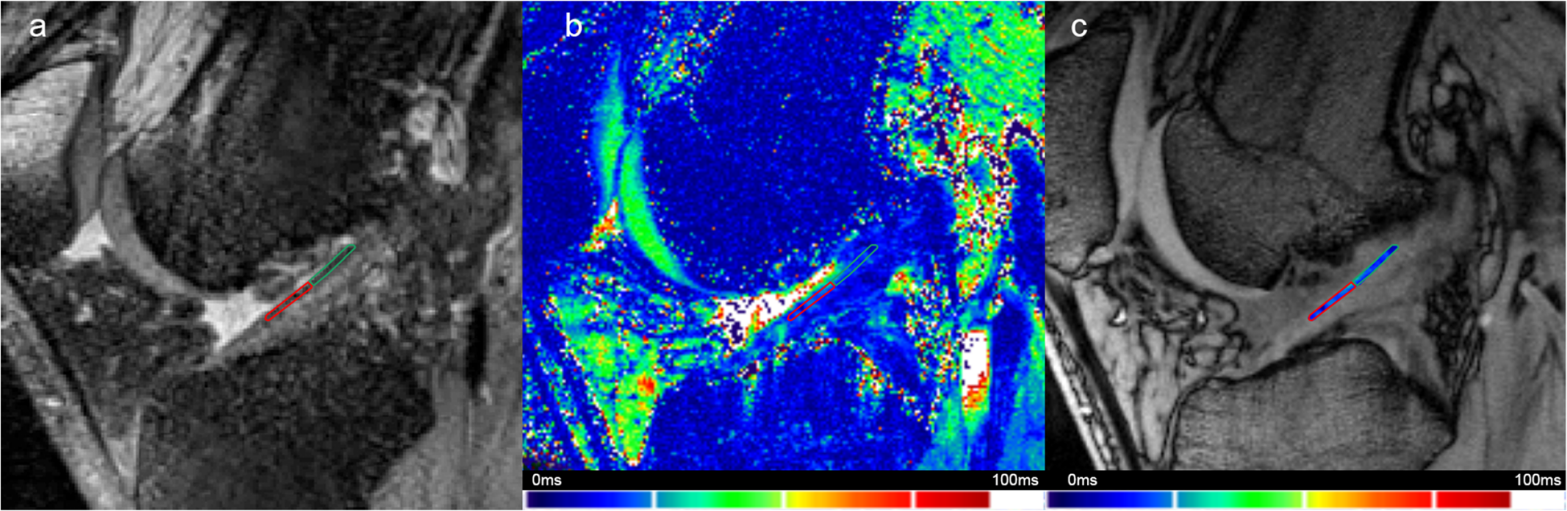
Demonstrative images showing a T2* map with regions of interest located into the anterior cruciate ligament (**a**), the corresponding colour-coded T2* map (**b**), and a T2*-weighted image with superimposed colour-coded T2* value overlay (**c**)

As only axial displacement and shear was expected, multimodal affine transformation was used to register T2* maps onto a corresponding high-resolution slice using an optimiser function (initial radius = 0.001; epsilon = 1.5e^-4^; growth factor = 1.01; maximum iterations = 300). All further maps were coregistered to the T2* map obtained during the first visit without load. The ROIs were placed onto a first-visit high-resolution slice and automatically transferred onto the other T2* maps. All automatically generated ROIs were visually inspected for artefacts characterised by uncommonly high T2* values (red spots on T2* maps). The corresponding voxels were manually excluded from further analysis. Figure 2 shows demonstrative images of the high-resolution three-dimensional TSE sequence with ACL ROIs, the corresponding T2* map, and a T2*-weighted image with superimposed colour-coded T2* value overlay. Using descriptive statistics, the means and standard deviations of T_2_* values were separately extracted for the central and femoral region. All image post-processing was performed using custom-made scripts written in MATLAB (64-bit version R2019a, The MathWorks Inc., Natick, MA, USA).

Due to the small sample (*n* = 3), individual and mean data are presented.

## Results

Individual T2* relaxation times by ACL region, pressure condition, and phase of the season are shown in Table 2.

**Table 2:** Individual T2* values (ms) by anterior cruciate ligament region, pressure condition, and phase of the season

	October		January		April		July	
	Central	Femoral	Central	Femoral	Central	Femoral	Central	Femoral
Measurements obtained at rest								
Subject 1	9.0 ± 3.4	8.2 ± 2.0	9.6 ± 2.6	8.0 ± 1.8	15.2 ± 3.4	13.9 ± 5.7	12.0 ± 3.1	11.5 ± 5.6
Subject 2	10.3 ± 3.3	10.3 ± 3.1	11.2 ± 2.3	12.7 ± 7.1	16.9 ± 4.1	13.1 ± 8.4	12.8 ± 2.9	15.5 ± 4.2
Subject 3	13.9 ± 2.6	10.1 ± 2.0	8.4 ± 2.7	8.6 ± 2.7	8.9 ± 2.2	8.9 ± 1.9	8.4 ± 2.4	10.3 ± 1.9
Measurements obtained during pressure application								
Subject 1	11.0 ± 2.6	8.9 ± 1.4	10.9 ± 2.3	9.3 ± 2.2	15.2 ± 3.1	11.2 ± 3.8	15.6 ± 3.0	12.3 ± 5.1
Subject 2	14.5 ± 3.6	11.3 ± 3.0	14.5 ± 1.8	16.4 ± 4.3	20.9 ± 6.7	13.5 ± 4.1	19.6 ± 4.9	15.7 ± 5.2
Subject 3	13.2 ± 3.5	14.0 ± 2.1	15.1 ± 2.5	10.1 ± 2.6	14.5 ± 2.2	9.4 ± 1.9	8.8 ± 2.1	9.6 ± 1.6

Data are mean ± standard deviation.

On average, baseline measurements of T2* at rest showed values of (mean value) 11.1 and 9.5 ms in the central and femoral ACL portion, respectively. While T2* values remained fairly stable between the measurements obtained in October and January, increases of 40.7% (central ACL portion) and 22.4% (femoral ACL portion) were observed between January and April. During the off-season (April–July), measures obtained in the central ACL region returned to baseline while they remained at an elevated level in the femoral portion.

**Fig. 3:**
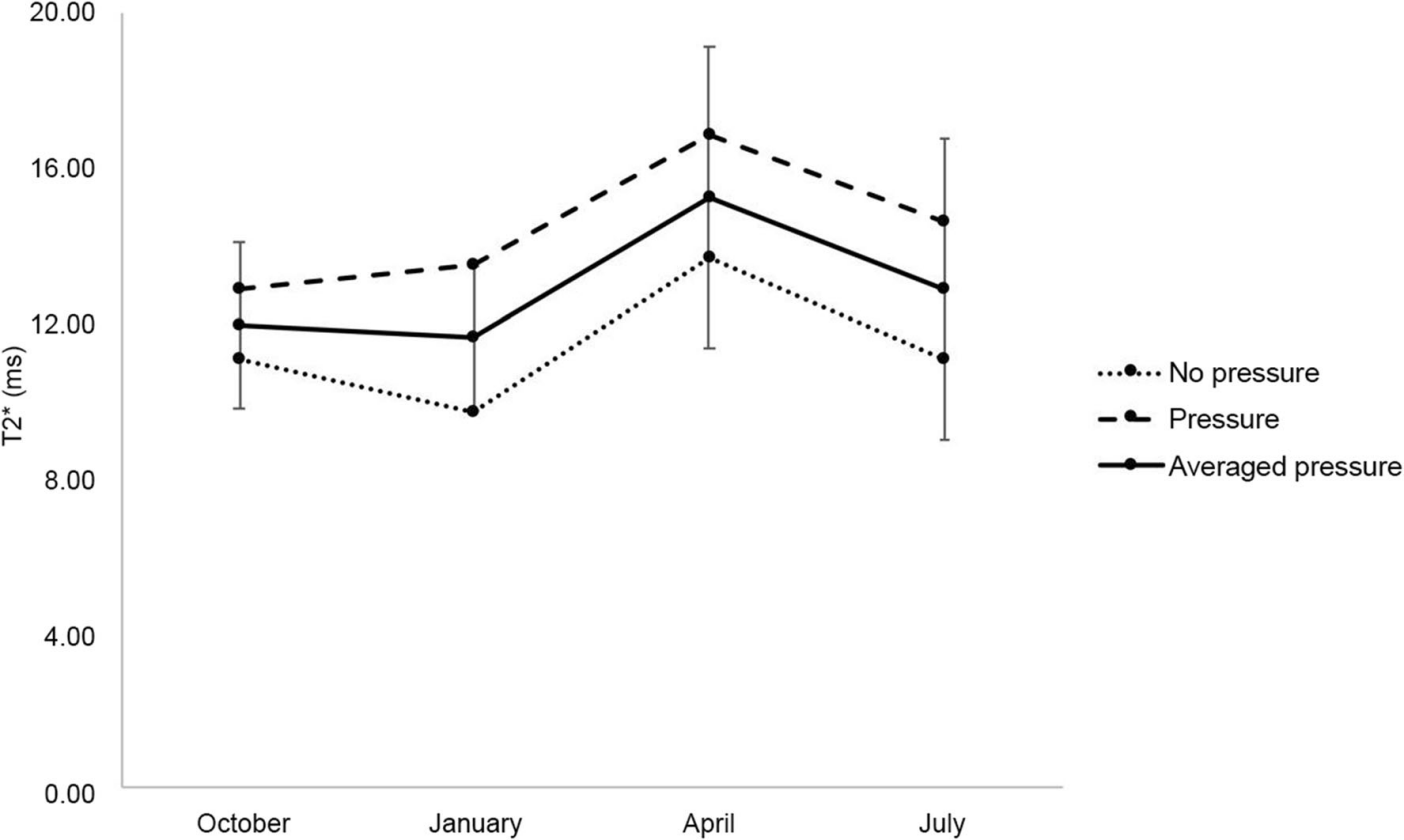
T2* values as measured in the central anterior cruciate ligament region averaged across subjects. Error bars are only shown for the average of measures obtained at rest and during pressure application (solid line in the middle)

During the application of pressure, T2* baseline mean values were 12.9 and 11.4 ms in the central and femoral ACL region, respectively. Just as in the measurements obtained at rest, T2* values in the central ACL region increased during the observation period, with the most prominent rises occurring between January and April (+ 24.6%). In the femoral region, by contrast, no substantial changes were observed in the same period (-4.6%). Figure 3 shows the seasonal changes in average T2* values as measured in the central ACL ROI at rest and during pressure application.

## Discussion

The development of noninvasive *in vivo* imaging-based biomarkers indicative of the ACL microstructure is of utmost clinical interest, as this might allow for conclusions about its mechanical properties and stress tolerance to be drawn. Such a tool would also be applicable to the assessment of the healing process following ACL reconstruction. One possible approach to obtaining such biomarkers that has received increased attention in the past decade relies on the MRI-based T2* mapping technique [12, 14–17]. T2* relaxation times are characteristic values that provide quantitative information about the tissue structure and composition, in particular the collagen fibre content and organisation, on a voxel-by-voxel basis. Aiming to provide evidence indicative of a weakening of the ACL structure that might promote fatigue tears and explain a potential accumulation of injuries in the later stages of the competitive season, we applied the T2* mapping technique to longitudinally study three professional alpine ski racers over the course of an entire season. The main finding of our pilot experiments is that T2* values as measured under different loading conditions peak in April, corresponding with the end of the competitive season in alpine ski racing.

In one of the first studies proposing T2* for the evaluation of the structure (and tensile properties) of the ACL, Biercevicz et al. [16] used a porcine model and found the volume and median greyscale in T2*-weighted images to predict the maximum failure load of patellar tendon allografts used for ACL reconstruction. Since signal intensities are sequence- and magnet-dependent [18], the same group then proceeded to refine the technique and use T2* relaxation times, which were found to correlate well with the results of tensile failure tests and the histologically assessed ligament maturity index of repaired minipig ACLs [14, 15]. According to these studies, lower T2* values coincide with greater stress tolerance and ligament maturity. Validation in human cadavers has proven to be more challenging, which may be due to the restricted variability of T2* relaxation times measured in cadaveric ligaments [17]. The joint body of evidence published to date, however, suggests that T2* mapping is a promising tool for the *in vivo* evaluation of ACL quality. This notion is also supported by a recent report testifying to excellent intra-rater (intraclass correlation coefficient 0.98) and inter-rater reliability (intraclass correlation coefficient 0.90) of T2* values measured in the ACL [19].

At rest, the T2* times measured in our study ranged between 8.4 and 16.9 ms in the central and between 8.0 and 15.5 ms in the femoral ACL region. These values agree well with those reported by Anz et al. [19] but are slightly lower than those found by Schmitz et al. [12], who reported ranges of 15.9–25.1 ms and 13.9–30.6 ms, respectively, in recreationally active men of similar age. Differences in the image acquisition protocol may explain the discrepant findings. Since Schmitz et al. [12] obtained the first echo only after 8.3 ms (the shortest TE used in our study was 3.7 ms), the shape of their signal intensity decay curve may have appeared with a different shape, thus yielding longer T2* times. Of note, our preliminary study was the first to measure T2* in the strained ACL. During the application of pressure, T2* values were greater by up to 33% and 20% in the central and femoral region of the ACL, respectively.

Stress MRI examinations have been performed in knee articular cartilage [20, 21]. These studies showed small, yet statistically significant load-associated decreases in T2 relaxation times, which were explained by cartilage compression and extrusion of water, leading to a more compressed collagen and proteoglycan structure. The stress tests performed in our study, by contrast, acted to extend the ACL. Thus, it is plausible to assume that the density of collagens and other solid ACL components was lower during pressure application, resulting in the observed increase in T2* values.

The primary findings of this study are the seasonal changes of T2* relaxation times in our sample of professional alpine ski racers that were most pronounced in the central region of the ACL and could be observed both at rest and during application of pressure. While average T2* values remained fairly constant between the measurements obtained in October and January, there was a marked increase of 41% (resting measurements) between January and April. Longer ACL T2* relaxation times have been associated with lower cell density and collagen organisation [15] and tensile load to failure [14]. Acknowledging the small sample size and explorative nature of the study, our data are, therefore, indicative of decreased ACL stress tolerance capacity in the end phase of the competitive season. The subsequent decrease of T2* values between April and July (-19%) might be interpreted as a partial recovery of the ACL structure. Further studies involving larger samples are required to substantiate these hypotheses.

This pilot study is subject to a number of limitations. First and foremost, the small sample size (*n* = 3) precluded us from performing inferential statistics, which is why our results must be considered preliminary. However, it should be pointed out that a substantial increase in T2* values between January and April was consistently observed in all three subjects. In addition, implausibly bright spots presumably resulting from magic angle artefacts [13] precluded the analysis of T2* values in the ACL proximal third. Furthermore, no direct measures of the ACL mechanical properties could be obtained, so the postulated correlation of T2* values with the ligament stress tolerance relies solely on findings from earlier studies in animals [14, 15]. Future studies might consider including stress imaging. Finally, it must be mentioned that, although T2* maps were coregistered with high-resolution images to warrant consistent in-plane ROI placement, minor differences due to not perfectly matched slices cannot be ruled out.

In conclusion, the results of this preliminary study show seasonal changes of T2* values mapped in the ACL of professional alpine ski racers, which are most pronounced in the ligament's central region. The values were found to peak in April, which coincides with the end of the competitive season and may reflect a decrease in stress tolerance, potentially increasing the risk of fatigue tears. Further research in larger samples is required.

## Data Availability

Data are available based on reasonable request from corresponding author.

## Abbreviations

ACL: Anterior cruciate ligament
GRE: Gradient echo
MRI: Magnetic resonance imaging
PD: Proton density
ROI: Region of interest
SPACE: Sampling perfection with application optimised contrasts using different flip angle evolutions
TE: Time of echo
TSE: Turbo spin echo
